# Identification of Metabolic QTLs and Candidate Genes for Glucosinolate Synthesis in *Brassica oleracea* Leaves, Seeds and Flower Buds

**DOI:** 10.1371/journal.pone.0091428

**Published:** 2014-03-10

**Authors:** Tamara Sotelo, Pilar Soengas, Pablo Velasco, Víctor M. Rodríguez, María Elena Cartea

**Affiliations:** Group of Genetics, Breeding and Biochemistry of Brassicas, Department of Plant Genetics, Misión Biológica de Galicia, Spanish Council for Scientific Research (MBG-CSIC), Pontevedra, Spain; Key Laboratory of Horticultural Plant Biology (MOE), China

## Abstract

Glucosinolates are major secondary metabolites found in the *Brassicaceae* family. These compounds play an essential role in plant defense against biotic and abiotic stresses, but more interestingly they have beneficial effects on human health. We performed a genetic analysis in order to identify the genome regions regulating glucosinolates biosynthesis in a DH mapping population of *Brassica oleracea*. In order to obtain a general overview of regulation in the whole plant, analyses were performed in the three major organs where glucosinolates are synthesized (leaves, seeds and flower buds). Eighty two significant QTLs were detected, which explained a broad range of variability in terms of individual and total glucosinolate (GSL) content. A meta-analysis rendered eighteen consensus QTLs. Thirteen of them regulated more than one glucosinolate and its content. In spite of the considerable variability of glucosinolate content and profiles across the organ, some of these consensus QTLs were identified in more than one tissue. Consensus QTLs control the GSL content by interacting epistatically in complex networks. Based on *in silico* analysis within the *B. oleracea* genome along with synteny with *Arabidopsis*, we propose seven major candidate loci that regulate GSL biosynthesis in the *Brassicaceae* family. Three of these loci control the content of aliphatic GSL and four of them control the content of indolic glucosinolates. GSL-ALK plays a central role in determining aliphatic GSL variation directly and by interacting epistatically with other loci, thus suggesting its regulatory effect.

## Introduction

The *Brassica* genus includes six agricultural important species which are grown in many countries, and important oil, condiment and vegetable crops. *Brassica* vegetables like broccoli, cabbage, Chinese cabbage, turnip greens and leaf rape, among others, are consumed throughout the world. FAO Statistics (FAOStat 2011) show that the production of cauliflower, broccoli, kales and other crucifers was 8.2% of the total vegetable production of the world in 2011. The most consumed crop of this genus in Europe and the USA is *Brassica oleracea*. This species includes cabbages, kales, broccoli and cauliflower, among others.

Glucosinolates (GSLs) are the major class of secondary metabolites found in the Brassicaceae falily, including the *Brassica* genus. The hydrolytic breakdown products of GSLs (especially isothiocyanates) have beneficial effects on human health, such as cytotoxic and apoptotic effects in damaged cells, thus preventing cancer in humans and reducing the risk for degenerative diseases [1]–[3]. They also enhance plant protection to abiotic and biotic stresses [4]. GSLs could exhibit certain adverse effects. For example, progoitrin can cause goiter in animals [5], which provoked the deliberate reduction of GSL levels in *B. napus* in the past. However, there is no evidence of any goitrogenic effect coming from *Brassica* consumption in humans [6]. Currently, efforts are concentrated on increasing the level of health promoting GSLs in *Brassica* crops. For example Sarikamis *et al.* [7] selected broccoli for higher levels of 3-methylsulphinylpropyl (GIB) and 4-methylsulphinylbutyl (GRA), which are the precursors of the isothiocyanates called iberin and sulforaphane, respectively. The beneficial effects of both isothiocyanates on human health are well known, having an influence on carcinogenesis during the initiation and promotion phases of cancer development [8]. Knowledge on the genetics underlying the synthesis and accumulation of GSLs in *Brassica* crops is an important tool for designing appropriate strategies in order to increase the content of those GSLs related to human health and plant protection.

**Figure 1:**
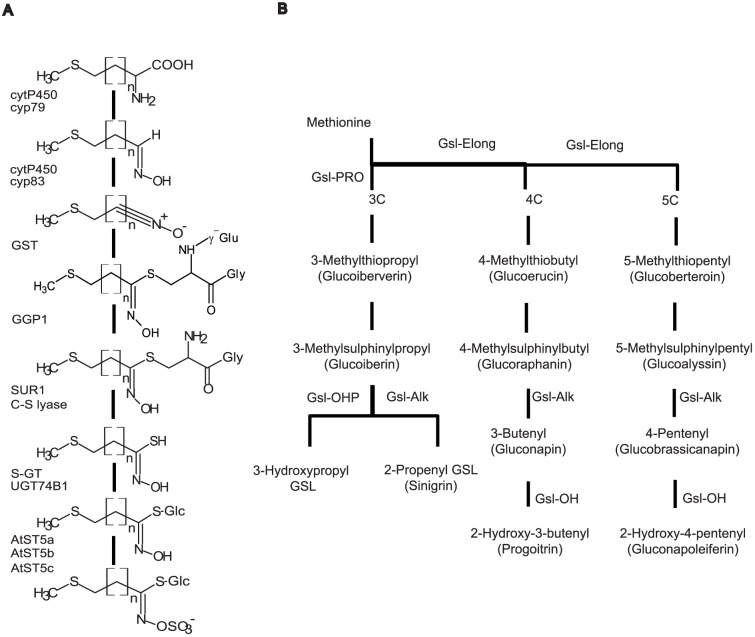
Formation of the core structure of the three major groups of glucosinolates in *A.thaliana*, including the genes controlling this process [11], [43]. (A). A biochemical genetic model of the biosynthesis of aliphatic glucosinolates in *Brassicaceae* including the major genes controlling this process [57] (B).

GSLs are divided into three different classes according to the amino acid precursor in biosynthesis: (1) aliphatic GSLs derived from alanine (Ala), leucine (Leu), isoleucine (Ileu), valine (Val), and methionine (Met); (2) aromatic GSLs derived from phenylalanine (Phe) and tyrosine (Tyr) and (3) indolic GSLs derived from tryptophan (Trp) [9]. In *Arabidopsis thaliana* and *Brassica* crops, most GSLs are synthesized from Met. GSL biosynthesis is a tripartite pathway involving three independent steps (Fig. 1A): (i) side chain elongation of some precursor amino acids such as Met and Phe, by adding one or several methylene groups. Chain elongation is carried out by methylthioalkylmalate synthase enzymes (MAM). (ii) Development of the core structure, which includes several steps: aldoxime formation catalyzed by the CYP79 family of cytochromes P450; aldoxime oxidation by the CYP83 family; thiohydroximic acid formation by conjugation to an S donor and after C-S bond cleavage; desulfoGLS formation by S-glucosyltransferase (S-GT); and GSL formation by sulfotransferase. (iii) Secondary modification of the amino acid side chain which includes oxidation, hydroxylation, methoxylation, desaturation, sulfation, and glycosylation [10], [11].

To date, major genes and transcription factors involved in the three steps of GSL biosynthesis have been identified and characterized in the model plant, *A. thaliana.* Based on *A. thaliana* homology, three loci were identified in *B. oleracea* and cloned [12]–[14]: two loci responsible for the elongation of the side chain of aliphatic GSLs named BoGSL-ELONG and BoGSL-PRO (homologous to MAM-1 and MAM-2 genes, respectively of *Arabidopsis*) and one locus responsible for side the chain desaturation and production of an alkenyl GSL named BoGSL-ALK (homologous to AOP2 gene of *Arabidopsis*). Afterwards, these loci, plus genes BoCS-lyase, BoGS-OH and BoCYP79F1, were mapped [15]. However, genes responsible for other steps of the metabolic pathway remain undiscovered. Identification of metabolic QTLs (QTLs) is essential for the understanding of the quantitative genetic control of secondary metabolites and it is an early step to identify the genes underlying trait variation. The high co-linearity between *A. thaliana* and *Brassica* species can be used in order to identify candidate genes underlying QTLs that affect GSL content. In addition to identifying structural and accumulation QTLs, it is important to determine the extent of epistatic interactions between loci which may play an important role in determining variability for GSL content.

The accumulation and profile of GSLs in plants are highly dependent on the genotype, although it is also affected by environmental and developmental factors. In *Arabidopsis*, GSL profiles have been systematically monitored during plant development and vary significantly among tissues and organs [16]–[19]. In *B. oleracea*, developmental stages and the type of tissues may modify the type of GSLs and its levels [20], [21]. Currently, little is known about the genetics of GSL content within the plant ontogeny. For this reason, it is necessary to develop a better understanding of the genetics underlying GSL biosynthesis and accumulation in different tissues in *B. oleracea*.

In the present study we identify QTLs for GSL composition and accumulation in *B. oleracea* leaves, flower buds and seeds in a double haploid (DH) population. We also perform a comparative genomic analysis based on *A. thaliana-B. oleracea* synteny in order to find candidate genes underlying QTL variation. Epistatic relationships among QTLs are also described. This information may increase the understanding on the quantitative genetic control of these traits and it is useful in order to identify genes controlling GSLs in *B. oleracea*.

## Materials and Methods

### Plant material and growing environments

A double haploid (DH) mapping population (BolTBDH) was employed in this work. The population was created from an F_1_ individual, from a cross between a DH rapid cycling of Chinese kale (TO1000DH3, P_1_) and a DH broccoli line ‘Early Big’ (P_2_) [22]. TO1000DH3 is the reference genome for the *B. oleracea* sequencing project. Firstly, parents and 155 DH lines were grown and selfed in the greenhouse in 2010 under: 16 h of daylight and a temperature of 24±2°C; 8 h of darkness having 18±2°C at night; and a relative humidity of 55% in order to obtain enough seed in the same environmental conditions. Selfing was carried out by bagging each individual plant inside a microperforated polyethylene bags. Five bulks of 10 mg of seed for each line were prepared for GSL analysis with the seeds obtained. In 2011 (from September to November), seeds from parents and 155 DH lines were sown with the same photoperiod and temperature as in 2010. Plants were sown in a completely randomized experiment with two replications and 4 plants per replication and DH line.

From each line, leave samples were taken at the 4 leaf stage and flower buds were taken differentially depending on the flowering time of each plant. One bulk was taken from each replication by mixing the four samples of leaves and flower buds. Samples were immediately frozen in liquid N_2_, transferred to the laboratory and conserved at –80°C until processing. All samples were lyophilized (BETA 2–8 LD plus, Christ) during 72 h. The dried material was powdered by using an IKA-A10 (IKA-Werke GmbH & Co.KG) mill, and the fine powder was used for GSL extraction.

### GSL identification and quantification

Sample extraction and desulfation were performed according to Kliebenstein *et al.* [23] with minor modifications. Three microliters of the desulfo-GSL extract for seeds and 5 μl for leaves and flower buds were used in order to identify and quantify GSLs. Chromatographic analyses were carried out on an Ultra-High-Performance Liquid-Chromatograph (UHPLC Nexera LC-30AD; Shimadzu) equipped with a Nexera SIL-30AC injector and one SPD-M20A UV/VIS photodiode array detector. The UHPLC column was a C18 Atlantis T3 waters column (3 µm particle size, 2.1×100 mm i.d.) protected with a C18 guard cartridge. The oven temperature was set at 30°C. Compounds were detected at 229 nm and were separated by using the following method in aqueous acetonitrile, with a flow of 0.8 mL min^–1^: 1.5 minutes at 100%H_2_O; a 11 min gradient from 0% to 25% (v/v) acetonitrile; 1.5 min at 25% (v/v) acetonitrile; a minute gradient from 25% to 0% (v/v) acetonitrile; and a final 3 min at 100% H_2_O. Data were recorded on a computer with the LabSolutions software (Shimadzu). Specific GSLs were identified by comparing retention times with standards and by UV absorption spectra.

GSLs were quantified at 229 nm by using sinigrin (SIN, sinigrin monohydrate from Phytoplan, Diehm& Neuberger GmbH, Heidelberg, Germany) and glucobrassicin (GBS, glucobrassicin potassium salt monohydrate, from Phytoplan, Diehm& Neuberger GmbH, Heidelberg, Germany) as external standards and expressed in µmol g^−1^ dry weight (DW). Calibration equations were made with, at least, five data points, from 0.34 to 1.7 nmol for sinigrin and from 0.28 to 1.4 nmol for glucobrassicin. The average regression equations for sinigrin and glucobrassicin were y  =  148818× (R^2^  =  0.99) and y  =  263822×(R^2^ =  0.99), respectively.

### Statistical analysis

A combined analysis of variance across organs and individual analyses of variance for each organ were made for individual and total GSL. Lines and organs were considered as fixed factors and replications were considered as random factors. Analysis of variance was performed with the PROC GLM of SAS [24].

The genetic map employed for the QTL analysis was created by Iñiguez-Luy *et al.* [22] having 279 markers (SSRs and RFLPs) distributed along nine linkage groups (C1-C9) with a total distance of 891.4 cM and a marker density of 3.2 cM/marker. Eight primer pairs described by Gao *et al*. [15] amplifying loci BoGSL-ELONG, BoGSL-ALK, BoGSL-PROa, BoGSL-PRO-b, BoCS-lyase, BoGS-OH, BoCYP79F1 and BoS-GT from *B. oleracea* were screened in parent DH lines. Besides, SSRs Gi12 Hasan *et al*. [25] and Ol12-D05 [26] were screened in parental DH lines. SSRs Gi12 and Ol12-D05 map in both sides of ATR1 gene of *A. thaliana* in chromosome 5 [25]. Amplifications were performed by following Gao *et al*. [15] and electrophoresis was carried out in 1% agarose gels and capillary electrophoresis system (CEQ 8000 Beckman, Coulter). Polymorphic markers were then screened in the BolTBDH mapping population, scored and assigned to linkage groups with JoinMap 3.0 sofware [27]. The threshold for assigning markers to linkage groups was a LOD score between 5 and 8.

Quantitative trait locus mapping was carried out thanks to a composite interval mapping method [28] by using the PLABQTL program [29]. In each organ (leaves, flower buds and seeds), analyses were carried out on each individual GSL and for each GSL type (aliphatic, indolic and aromatic) as well as on the total GSLs. A likelihood odds (LOD) threshold of 3.2 was chosen in order to declare a putative QTL significant by following the method described by Van Ooijen [30]. The confidence intervals were set at 95%. The analysis and cofactor election were carried out by following PLABQTL’s recommendations, by using an ’F-to-enter’ and an ’F-to-delete’ value of 7.

The proportion of phenotypic variance explained for a specific trait was determined by the adjusted coefficient of determination of regression (R^2^) fitting a model including all detected QTLs [31]. Fivefold cross-validation of QTLs was performed by following the procedures described by Utz *et al*. [32]. The frequency of QTL detection gives us an estimation of the precision of QTL localization.

Significant QTLs for individual GSLs were integrated by using a QTL meta-analysis with BioMercator 2.1 software in order to give consensus QTLs [33]. An Akaike-type statistical criterion (AIC value) indicated the model which best fitted the data, including the number and the consensus QTLs positions. The aim of performing a meta-analysis was to find if a genomic region could determine the GSL content of different GSLs and if the same QTL was present in the three organs under study.

Iñiguez-Luy *et al*. [22] identified collinear genomic blocks between the BolTBDH mapping population and *A. thaliana* by using a synteny analysis. This information was employed in order to identify candidate genes that may directly account for GSL QTLs in *B. oleracea.* In following this approach, we tried to locate 46 genes involved in GSL metabolism in *A. thaliana* which were obtained from TAIR (The *Arabidopsis* Information Resource) on the BolTBDH map by *in silico* mapping.

Epistatic interaction analysis among QTLs was performed by using the R/qtl package of the R software [34].

## Results

### Phenotypic variation in GSL content

Twelve GSLs, belonging to three chemical classes, were detected in the BolTBDH population (Table 1). Eight GSL were aliphatic, three of them belonging to the 3C group: 3-methylthiopropyl (GIV), 3-methylsulfinylpropyl (GIB) and 2-propenyl (SIN); four belonging to the 4C group: 4-methylthiobutyl (GER), 4-methylsulfinylbutyl (GRA), 3-butenyl (GNA) and 2-hydroxy-3-butenyl (PRO); and one belonging to the 5C group: 5-methylsulfinylpentyl (ALY). Three indolic GSLs: 4-hydroxy-3-indolylmethyl (OHGBS), 3-indolylmethyl (GBS); and 1-methoxy-3-indolylmethyl (NeoGBS), and one aromatic GSL, 2-phenylethyl (GNT), were also detected.

**Table 1:** Glucosinolate (GSL) profiles and concentrations (µmol g-1dw) of parents and mean and range of the DH population.

	Leaves				Flower buds				Seeds			
GSL	P_1_	P_2_	Population mean (range)	Population %	P_1_	P_2_	Population mean (range)	Population %	P_1_	P_2_	Population mean (range)	Population %
GIV	-	-	-	-	-	-	-	-	0.53	0.00	1.63 (0–6.81)	1.39
GIB	0.00	0.00	0.29 (0–1.10)	5.33	0.00	0.00	0.89 (0–3.40)	6.60	1.04	0.00	6.06 (0–41.20)	5.14
SIN	2.42	0.00	0.44 (0–1.57)	8.02	1.57	0.00	1.22 (0–4.51)	9.04	42.32	0.00	8.15 (0–46.82)	6.91
GER	-	-	-	-	0.00	0.20	0.18 (0–0.50)	1.30	0.54	7.27	8.25 (0.27–34.54)	6.99
GRA	0.00	0.45	0.97 (0–6.65)	17.63	0.21	5.14	3.64 (0.15–17.35)	26.93	0.72	21.69	22.62 (0.48–74.14)	19.17
GNA	3.56	0.00	0.86 (0–6.38)	15.64	3.09	0.00	3.12 (0–17.12)	23.02	77.31	0.00	44.50 (0–138.40)	37.72
PRO	0.00	0.00	0.56 (0–2.77)	10.19	0.51	0.12	1.12 (0–13.22)	8.28	0.94	0.00	20.45 (0–129.80)	17.33
ALY	-	-	-	-	-	-	-	-	0.00	0.00	0.25 (0–2.38)	0.22
OHGBS	0.00	0.00	0.034 (0–0.36)	0.62	0.00	0.09	0.13 (0–0.41)	0.98	4.80	1.66	4.34 (1.81–10.20)	3.68
GBS	0.68	1.30	1.02 (0.005–3.24)	18.50	0.35	0.52	0.97 (0.14–3.87)	7.17	0.00	0.40	0.75 (0–5.37)	0.64
NeoGBS	1.72	2.34	1.14 (0.069–6.39)	20.63	0.59	1.06	1.86 (0.13–11.84)	13.78	0.53	0.37	0.50 (0–1.70)	0.43
GNT	0.19	0.79	0.19 (0–0.79)	3.44	0.18	0.86	0.39 (0–1.15)	2.90	0.38	0.21	0.42 (0–1.39)	0.36
Aliphatic	5.97	0.65	1.58 (0–6.97)	40.20	5.38	5.29	6.63 (0.59–20.98)	67.70	123.70	28.97	77.78 (30.38–157.15)	93.34
Indolic	2.40	3.65	2.17 (0.09–8.47)	55.21	0.94	1.68	2.88 (0.46–12.14)	28.57	5.33	2.44	5.29 (2.12–10.29)	6.36
Aromatic	0.19	0.79	0.19 (0–0.79)	4.22	0.18	0.86	0.39 (0–1.15)	3.86	0.38	0.21	0.42 (0–1.39)	0.50
Total	8.56	5.09	4.01 (0.12–13.20)	100.00	6.50	7.99	10.13 (1.47–24.56)	100.00	129.41	31.61	83.33 (36.23–160.29)	100.00

P_1_, DH rapid cycling of Chinese kale (TO1000DH3); P_2_DH broccoli line ‘Early Big’; Aliphatic glucosinolates: GIV, Glucoiberverin; GIB, Glucoiberin; SIN, Sinigrin; GER, Glucoerucin; GRA, Glucoraphanin; GNA, Gluconapin; PRO, Progoitrin; ALY, Glucoalyssin; GBN, Glucobrassicanapin; Indolic glucosinolates: OHGBS, 4-hydroxyglucobrassicin; GBS, Glucobrassicin; NeoGBS, Neoglucobrassicin; Aromatic glucosinolate: GNT, Gluconasturtiin.

Different GSL profiles were detected in the parental lines. The following aliphatic GSLs were found in P_1_ (TO1000DH3) in different organs: GIV, GIB, SIN GER, GRA, GNA, and PRO. Aliphatic GER and GRA and PRO were detected in P_2_ (‘Early Big’ broccoli) meantime aliphatic ALY was found in the mapping population but it was not detected in its parents. Therefore, 3C and 4C GSLs were found in P_1_, while only 4C GSLs were found in P_2_. Alkenyl GSLs (SIN, GNA and PRO) were found in P_1_ but not in P_2_ (only trace amounts of PRO in flower buds) (Table 1).

The GSL profile of the mapping population varied depending on the organ. In leaves, 55.2% of GSLs were indolic and 40.2% of GSLs were aliphatic, being NeoGBS and GRA the major GSLs respectively. In seeds, 93.3% of total GSLs were aliphatic, and GRA, GNA and PRO were the major GSLs. The GSL profile of flower buds was intermediate among leaves and seeds as 67.7% of total GSLs were aliphatic and 28.6% were indolic. GRA, GNA and NeoGBS were the major GSLs in this organ. GIV and ALY were exclusively found in seeds, meanwhile GER was only found in flower buds and seeds (Table 1).

Aliphatic GSL content in P_1_ was higher than that found in P_2_ in the three organs analyzed (Table 1). SIN and GNA were the major aliphatic GSLs found in the three organs for P_1._ In contrast GRA was the major GSL in P_2_ in the three organs. Regarding indolic GSLs, GBS and NeoGBS were found as the most abundant in both parents in both leaves and flower buds, while OHGBS was the major GSL found in seeds. Indolic GSL content was higher in P_2_ compared to P_1_ in both leaves and flower buds.Total GSL content in P_1_ was higher than that found in P_2_ leaves and seeds (Table 1).

In the mapping population, the content of individual GSLs as well as the content of aliphatic, indolic and total GSLs showed continuous distributions. Extreme phenotypes were found for all traits, with the exception of GNT in leaves, compared to phenotypes observed in parent lines (Table 1). For example, extreme mean values of some individual GSL content in the mapping population are far beyond the content of any of the parents. For instance, GRA content in seeds was 0.72 µmol g^−1^dw in P_1_ and 21.69 µmol g^−1^dw in P_2_. The average GRA content in the mapping population was 22.62 µmol g^−1^dw and ranged from 0.48 to 74.14 µmol g^−1^dw (Table 1). Total GSL content in the different organs varied nearly 18-fold within the mapping population. The average content of total GSLs was 4.01 µmol g^−1^dw in leaves, 10.13 µmol g^−1^dw in flower buds and 83.3 µmol g^−1^dw in seeds (Table 1).

### Analysis of variance

Significant organ x line interactions were found for all traits, therefore individual analyses were carried out by organ. The source of variation due to lines was highly significant for the most traits, except ALY and OHGBS in leaves and GIV and NeoGBS in seeds. The source of variation due to replications was in most cases non significant (data not shown).

### QTL analysis

**Figure 2:**
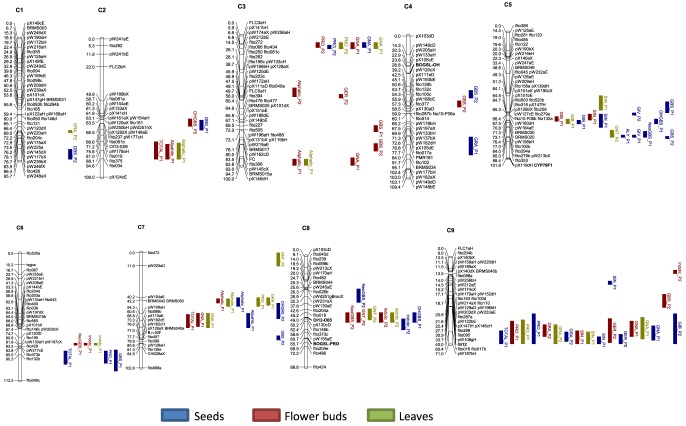
Framework map of DH population showing eighty-two metabolic quantitative trait loci (QTL) for individual GSLs and sums of GSLs. Linkage groups were labeled by following the nomenclature of Iñiguez-Luy *et al.* [22]. Bars represent the LOD confidence interval of each QTL. QTLs are in different colors depending on the plant organ: leaves (green), flower buds (red) and seeds (blue). After the name of each QTL, -P_1_ indicates allele from DH rapid cycling of Chinese kale (TO1000DH3) and -P_2_ indicates allele from DH broccoli line ‘Early Big’.

Three out of eight primer pairs designed by Gao *et al*. [15] were polymorphic in of the mapping population’s parents. These markers could be mapped and located in three different linkage groups. BoGSL-OH mapped on C4 (28.8 cM), BoCYP79F1 mapped on C5 (102 cM) and BoGSL-PROb mapped on C8 (66 cM). SSRs OL12-D05 and Gi12 were also polymorphic and they mapped on C8 (49 cM) and C9 (40 cM), respectively. QTL analyses were carried out with 279 markers designed by Iñiguez –Luy and the five newly mapped primer pairs. No significant QTL was detected in any of the map positions where BoGSL-OH, BoCYP79F1 and BoGSL-PROb were located (Fig. 2).

Eighty-two significant QTLs were detected being spread all over the 9 linkage groups of *B. oleracea*. The number of QTLs by linkage group ranged between two in C1 and 19 in C9 (Fig. 2). Twenty significant QTLs were found in leaves. The value of R^2^ ranged between 10.3% for GNA in C7 and 34.3% for the sum of aliphatic GSLs in C7 (Table S1). Half of QTLs had a frequency of cross-validation higher than 50%. Twenty-nine significant QTLs were detected in flower buds. R^2^ value ranged between 10.4% for the sum of aliphatic GSLs in C3 and 49.7% for the sum of aliphatic GSLs in C9, respectively. Eighteen QTLs had a frequency of cross-validation higher than 50%. Thirty-three significant QTLs were found in seeds. R^2^ value varied between 10.3% for the sum of indolic GSLs in C6 and 49.4% for ALY in C5. Twenty-eight QTLs had a frequency of cross-validation higher than 50%.

### Consensus QTLs

Based on the position of the QTLs and taking into account their confidence interval, a meta-analysis in order to render consensus QTLs for GSL concentration was carried out. Eighteen consensus QTLs were detected (Table 2). Fourteen consensus QTLs were present in seeds, 12 QTLs in leaves and 14 QTLs in flower buds. Seven QTLs were common to flower buds, leaves and seeds; three QTLs were exclusively found in leaves, two QTLs were exclusively found in flower buds and other two QTLs were exclusive found in seeds. In order to make the discussion clearer, results regarding consensus QTLs are going to be presented according to each chemical GSL class.

**Table 2:** Position and characteristics of consensus QTLs found in BolTBDH mapping population.

				Aliphatic																		
				3C			4C				5C			Indolic				Aromatic	Total	Plant organ		
LG	No	Peak position (cM)	Confidence interval (cM)	GIV	GIB	SIN	GER	GRA	GNA	PRO	ALY	GBN	Sum of aliphatic GSLs	OHGBS	NeoGBS	GBS	Sum of indolic GSLs	GNT	Total GSL	Seeds	Leaves	Flower buds
2	2.1	67.55	62.3–78.9											x		x				x		x
	2.2	89.74	83.7–95.7										x						x		x	x
3	3.1	7.09	4.9–9.3						x	x										x	x	x
	3.2	46.6	36.4–56.8				x						x									x
	3.3	79.1	69.0–89.2													x						x
	3.4	99.3	92.6–105.9						x				x								x	x
4	4.1	47.3	37.1–57.4													x				x		x
	4.2	84.0	70.9–97.0					x												x		
5	5.1	70.2	68.3–72.0	x	x	x								x		x				x	x	x
	5.2	84.1	81.9–86.3	x							x				x	x		x		x	x	
6	6.1	86.8	81.9–91.7							x					x	x	x		x	x	x	x
7	7.1	2.0	0–17.8															x			x	
	7.2	42.9	40.3–45.4					x		x			x							x	x	x
	7.3	56.4	52.3–60.5						x				x	x					x	x	x	x
	7.4	76.0	67.7–84.2													x				x		
8	8.1	45.8	41.5–50.1											x	x		x	x		x	x	x
9	9.1	27.0	20.7–33.3			x										x	x			x		x
	9.2	64.9	63.2–66.6		x	x	x	x	x	x									x	x	x	x

Aliphatic glucosinolates: GIV, Glucoiberverin; GIB,Glucoiberin; SIN, Sinigrin; GER, Glucoerucin; GRA,Glucoraphanin; GNA, Gluconapin; PRO, Progoitrin; ALY, Glucoalyssin; GBN, Glucobrassicanapin. Indolic glucosinolates: OHGBS, 4-hydroxyglucobrassicin; GBS, Glucobrassicin; NeoGBS, Neoglucobrassicin. Aromatic glucosinolate: GNT, Gluconasturtiin.

### Aliphatic GSLs

Located in C3, consensus QTL-3.1 controls the content of PRO and GNA in the three organs (Table 2). Alleles for increasing PRO content are given by P_1_, while alleles for increasing GNA content are given by P_2_ (Fig. 2). Consensus QTL-5.1, located in C5, controls the content of GIB and SIN in the three organs. Alleles for increasing the content of both GSLs are given by P_1_. In C9, consensus QTL-9.2, which controls the content of PRO, GNA, GRA, GER (4C-GSL) and SIN, and GIB (3C-GSL) in the three organs, was located. Alleles for synthesis of PRO, SIN and GNA are given by P_1_, while alleles for increasing the content of GRA, GER and GIB are given by P_2_ (Fig. 2). Other QTLs which control aliphatic GSL content exclusively are QTL-1.1, QTL-2.2, QTL-3.1, QTL-3.2, QTL-3.4, QTL-4.2 and QTL-7.2.

### Indolic and aromatic GSLs

Several consensus QTLs only controlled the indolic GSL content. QTL-1.2, QTL-3.3, QTL-4.1 and QTL7.4 determined the GBS content in seeds and flower buds (Table 2). Alleles for increasing the content of GBS are given by P_2_ in all these QTLs except for QTL-3.3, where alleles came from both parents. Consensus QTL-2.1 determines the content of OHGBS and GBS in seeds and flower buds. The allele for increasing OHGBS is given by P_2_ in flower buds, while the allele for increasing GBS content is given by P_1_. Consensus QTL-8.1 determines the OHGBS, NeoGBS and total indolic GSL content in the three organs. Besides, this QTL also controls the content of the aromatic GNT. Other QTLs for GNT content are QTL5.2 and QTL7.1. The genomic regions QTL-1.2, QTL-2.2 and QTL-7.4 are collinear with genomic regions of *A. thaliana* in chromosomes 4, 5 and 2. In these regions, genes CYP83B1, CYP81F2 and CYP79B3 from *A. thaliana* were found by means of *in silico* mapping.

### Epistatic networks

A total of 85 significant epistatic interactions were found when taking into account the three organs and all the traits. Thirteen epistatic interactions were found in leaves, 52 in flower buds and 13 in seeds. Some of these interactions are common to the three organs under study. Sixty-eight interactions were detected in aliphatic GSLs, 13 in indolic GSLs and 4 in total GSLs. An average of 3.5 significant epistatic interactions was found per trait (Fig. S1).

**Figure 3:**
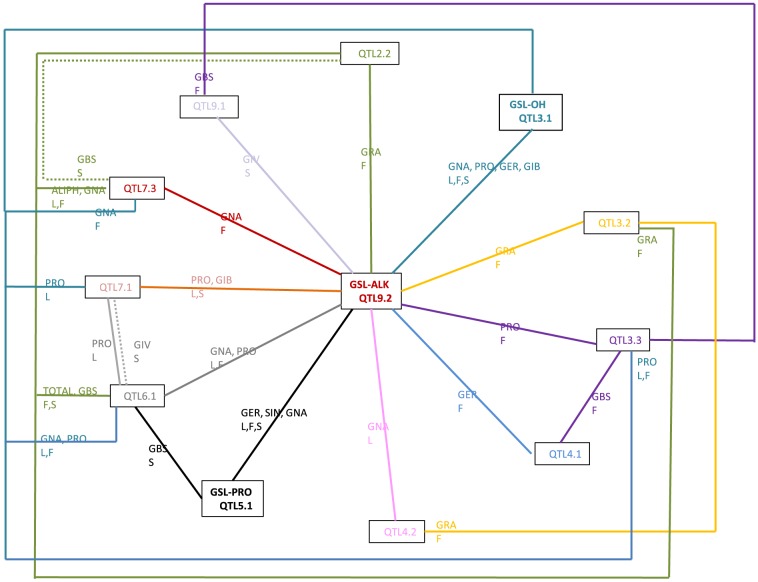
An epistatic network including all the significant relationships of QTL9.2 (GSL-ALK) with other QTLs. Aliphatic glucosinolates: GIV, Glucoiberverin; GIB, Glucoiberin; SIN, Sinigrin; GER, Glucoerucin; GRA, Glucoraphanin; GNA, Gluconapin; PRO, Progoitrin; ALY, Glucoalyssin; GBN, Glucobrassicanapin; ALIPH: sum of aliphatic GSLs; Indolic glucosinolate: GBS, Glucobrassicin; TOTAL: sum of total GSLs. Organs: L, Leaves; F: Flower buds; S: seeds. Continuous lines represent positive epistatic interactions while dashed lines represent negative epistatic interactions.

Forty-two interactions were detected between QTLs, being two of them negative. Twenty interactions were detected between QTL9.2 (proposed as GSL-ALK in this work) and other QTLs in traits related to aliphatic GSLs (Fig. 3). The relationship between QTL9.2 and QTL 3.1 (proposed as GSL-OH) was found for the aliphatic GNA, PRO, GER and GIB in the three organs under study. The relationship between QTL9.2 and QTL5.1 (proposed as GSL-PRO) was found for the aliphatic GER, SIN and GNA in the three organs (Fig. 3). In the network controlled by GSL-ALK, interactions between aliphatic and indolic QTLs were observed. For example, QTLs 3.3, 4.1 and 9.1 control the GBS content and the three of them interact with QTL 9.2 in order to produce aliphatic GSLs (Fig. 3).

## Discussion

### Phenotypic variation in GSL content

Parents of the mapping population had different GSL profiles and concentration. Particularly, parent P_2_ has a higher concentration of GRA and a lower concentration of GNA than parent P_1_ in the three organs. GRA is found in several *B. oleracea* crops like cauliflower, cabbage and kale, although high levels of GRA equivalent to those found in P_2_ (‘Early Big’ broccoli) are always found in broccoli [35]–[38]. The effect of sulforaphane, the isothiocyanate derived from GRA, against cancer has been reviewed in detail [8], [39]. As a result of these epidemiological and biomedical studies, GRA is now viewed as a quality trait in *B. oleracea* crops to be targeted in breeding programs.

Distributions of individual and sums of GSLs were in most cases transgressive. These types of segregations have been described before for GSL content in *Brassica* [40], [41] and could be due to new combinations of additive alleles or to epistatic interactions among loci for GSLs, which have already been described [42], [43].

Total GSL content varied considerably depending on the organ under study. As it was expected, seeds accumulated the highest GSL content followed by flower buds and leaves. After studying the GSL content in different organs of *A. thaliana*, Brown *et al*. [17] found that seeds had the highest concentration followed by inflorescences, siliques, leaves and roots. Velasco *et al*. [20] found that the GSL content in flower buds was higher than kale leaves [20]. These results may reflect the need to indicate *de novo* synthesis of GSLs and/or mobilization [17].

The GSL profile also varied considerably depending on the organ. In fact, seeds were mostly composed of aliphatic GSLs, whereas indolic GSL were predominant in leaves. Flower buds had an intermediate profile. Besides, flower buds and seeds showed more diversification of aliphatic GSLs, since GIV and ALY were only found in seeds and GER was only found in flower buds and seeds. Agreeing with these results, kale leaves are characterized by high amounts of indolic GSLs during the first plant stages, while aliphatic GSLs are predominant in flower buds and in leaves taken at the end of the vegetative stage [20]. A similar pattern was observed in *A. thaliana*, where seeds are distinguished by unique aliphatic constituents and low level of indolic compounds. After germination, the proportion of aliphatic GSLs declined with age, thus resulting in the predominance of indolic GSLs by the time of senescence [17].

### QTLs analysis

Seven out of 20 consensus QTLs determined the content exclusively in one of the three organs under study. Our results suggest that the regulation of genes underlying several QTLs is organ-dependent. Feng *et al*. [43] analysed QTLs for GSL content in leaves and seeds of *B. napus* and found 17 QTLs which were exclusively detected in leaves. Kliebenstein [23] found three organ-specific QTLs for aliphatic GSLs in both leaves and seeds of *A. thaliana.* A similar number was found for indolic GSLs.

### Aliphatic GSLs

Several major loci determine the profile and content of aliphatic GSLs in *Brassica* [44]. The GSL-ELONG and GSL-PRO loci regulate the side chain length (Fig. 1B). The presence of 4C-GSL is controlled by a dominant allele of GSL-ELONG (GSL-ELONG+), whereas the presence of 3C-GSL is controlled by a dominant allele of GSL-PRO (GSL-PRO+) [45]. GSL-ALK controls side chain desaturation. The presence of GSL-ALK+ in 3C-GSL determines the production of alkenyl GSL. GSL-OHP catalyzes production of 2-hydroxypropyl GSL, but this GSL was not detected in parents or the mapping population. GSL-OH controls PRO production and its action is conditioned by the presence of GSL-ALK+ [45]. After analyzing parents of the mapping populations, it can be concluded that the genotype of P_1_ is GSL-ELONG+, GSL-PRO+, GSL-ALK+ and GSL-OH+, while the genotype of P_2_ is GSL-ELONG+, GSL-PRO-, GSL-ALK-. Because P_2_ is GSL-ALK- and the presence of GSL-ALK+ is needed in order to produce hydroxylated GSL, the genotype for the locus GSL-OH could not be determined. GSL-ELONG cannot be located into the mapping population, because both parents had the same genotype for this locus. Primer pairs amplifying loci GSL-PROb and GSL-OH designed by Gao *et al*. [15] were located in the mapping population in different positions as those reported by the authors, thus probably indicating an unspecific amplification of PCR products.

Consensus QTL-5.1 controls the amount of three 3C-GSLs: GIB, GIV and SIN. Alleles for increasing 3C-GSLs content are given by P_1_. Thus, GSL-PRO would be a good candidate gene for this QTL. This major locus was cloned [14] and mapped at the top of C5 in *B. oleracea* [15]. Position of C5 markers in the map of Iñiguez-Luy *et al*. [22] is inverted with regard to C5 in the map of Gao *et al*. [15]. Taking this into account, the position of QTL-5.1 coincides with that of GSL-PRO. This information together supports the validation of the candidate gene. This QTL also controls the content of two indolic GSLs GBS and NeoGBS. Aliphatic and indolic GSLs are synthesized and subsequently modified by two independent parallel pathways [46]. However, there are cross-talks between both pathways. Wentzell *et al*. [46] found that *GSL.INDOLIC.IV.8* and *GSL.INDOLIC.V.20* QTLs, which control the content of several indolic GSLs in *A. thaliana*, map in the same genomic locations as GSL-AOP and GSL-ELONG loci which control aliphatic GSLs [46].

Consensus QTL-9.2 controls the amount of several GSLs. Alleles for increasing alkenyl GSL content (SIN, PRO, GNA) are given by P_1_, while alleles for increasing non alkenyl GSL content (GRA, GER, GIB) are given by P_2_ (Fig. 1B). Locus GSL-ALK was studied and cloned by Li and Quiros [13] and mapped in C9 [15] in the same position as QTL-9.2. Consensus QTL-3.1 controls the amount of GNA and its hydroxylated form PRO (Fig. 1B). Curiously, alleles for increasing GNA content are given by P_1_which is GSL-OH+, while alleles for increasing PRO content are given by P_2_. This makes us think that P_2_ is also GSL-OH+. The function of this QTL would correspond to gene GSL-OH. Gao *et al*. [15] mapped this gene in C9, close to GSL-ALK. The position of the gene does not correspond to QTL-3.1. After searching in the whole genome sequence of *B. rapa*, Zang *et al*. [47] and Wang *et al*. [48] found GSL genes homologous to those of *A. thaliana*. Three different copies of gene GSL-OH were found in *B. rapa* due to the triplicate nature of its genome [48]. Several copies of the same genes could also exist in *B. oleracea*.

During the first stage of the development of the core structure of aliphatic GSL (Fig. 1), the gene CYP79F1 metabolizes mono- to hexahomomethionine into their corresponding aldoxime in *A. thaliana* [49]. Primers designed in order to amplify this gene in *B. oleracea* [15] were employed in this work. CYP79F1 mapped in C5, in the same position found by Gao *et al*. [15], but no QTL was found in this position, thus indicating that both parents have the same allele for this gene. Consensus QTL-2.1 controls the content of total aliphatic GSLs in leaves and flower buds and the total GSL content in flower buds, but it does not control the content of any individual GSL, thus suggesting that the gene underlying this QTL may have a regulatory role in the aliphatic GSL pathway. Two R2R3-Myb transcription factors (Myb 28 and Myb 29) positively control biosynthesis of aliphatic GSLs in *A. thaliana* [50] and could be candidate genes for this consensus QTL.

### Indolic and aromatic GSLs

In the first stage of the development of the core structure (Fig. 1A) of indolic GSLs, two cytochromes P450 (CYP79B2 and CYP79B3) catalyze the conversion of Trp to indole-3-acetaldoxime in *A. thaliana* [51], [52]. Overexpression of CYP79B2 results in an increased accumulation of indole GSLs, specifically 3-indolylmethyl (GBS) and 4-methoxy-glucobrassicin (MeOH-GBS) (not detected in this work). In the next step, CYP83B1 catalyzes the transformation of indole-3-acetaldoxime into to *S* -alkyl-thiohydroximate (Fig. 1A) [53], [54]. The Myb transcription factor ATR1 from *A. thaliana* regulates the expression of genes CYP79B2, CYP79B3, and CYP83B1. Overexpression of ATR1 leads to lines with higher levels of total indolic GSLs than wild-type plants [55]. CYP81F2 catalyzes the hydroxylation at position 4 of the indole ring of GBS, which results in the formation of OHGBS and MeOH-GBS [56].

After *in silico* mapping of *A. thaliana* GSL genes, CYP79B2 and CYP79B3 were located inside the confidence interval of consensus QTL-1.2 and QTL-7.4. Both of them determine variation for GBS in seeds, agreeing with a possible high expression of candidate genes CYP79B2 and CYP79B3.

SSRs Gi12 and Ol12-D05 map in both sides of ATR1 gene of *A. thaliana* in chromosome 5 [25]. Gi12 mapped in C9 in our work, where no QTL was detected. Ol12-D05 mapped within the consensus QTL-8.1confidence interval. This QTL determines variation for OHGBS, NeoGBS and total indolic GSL content in the three organs analyzed.

The high apparition of QTLs for indolic GSL content agrees with a high expression of ATR1 candidate gene. Besides, aromatic GNT is also controlled by this QTL. Aromatic GSLs are also a substrate of CYP83B1, regulated by ATR1. These results together suggest that ATR1 could be a possible candidate gene for QTL-8.1.

Consensus QTL-2.1 determines variation for NeoGBS and GBS in flower buds and seeds. Candidate gene CYP81F2, metabolizing the step from GBS to NeoGBS from *A. thaliana,* was found in the confidence interval of this QTL.

The *B. oleracea* whole genome sequencing is currently carried out by using TO1000DH3 as the reference genome. Sequences are being aligned by using mapping population BolTBDH. *B. oleracea* sequencing project will be a great opportunity to link sequences with the QTLs described in this work.

### Epistatic networks

Significant epistatic interactions were found for the three organs under study. On the contrary of what was found by *Feng et al.* [43] in *B. napus*, part of the interactions were common among organs. The number of interactions was higher in flower buds, thus indicating a more complex regulation of GSL biosynthesis in this organ. Epistatic interactions for indolic GSLs were less complex than for aliphatic GSLs. 49% of the epistatic interactions detected were between QTLs, thus indicating that variability for GSLs content is determined directly by QTLs and indirectly by interacting with other loci.

Epistatic interactions among GSL-ALK, GSL-PRO and GSL-OH, determine variability for aliphatic GSL content and have been described before (reviewed by Kliebenstein [44]) in *A. thaliana*. They are mediated by transcriptional factors. In this work we have found that GSL-ALK plays a central role in the network of epistatic interactions for aliphatic GSLs, suggesting a possible regulatory effect of this locus. Indirectly, GSL-ALK also controls the variability for the indolic GSL named GBS, thus indicating cross-talk between indolic and aliphatic pathways. This information supports the results found by Wentzell *et al.* [46] in *A. thaliana*. These authors transformed a null accession for AOP2 and AOP3 genes (GSL-ALK locus) with AOP2 gene from *B. oleracea*, thus resulting in the production of alkenyl GSLs, doubling of total aliphatic GSL content and the induction of aliphatic GSL biosynthetic genes and regulatory genes.

## Conclusions

An extensive analysis of QTLs controlling GSL variation in three different organs of *B. oleracea* has been presented. Possible candidate genes for different QTLs have been proposed based on the phenotypic study of the progeny and on the synteny with *A. thaliana*. Epistatic interactions among QTLs have been detected showing a central role of GSL-ALK in determining aliphatic GSL variation and suggesting a regulatory effect of this locus. Further work is going to be carried out in order to validate them and to find new candidate genes for remaining QTLs.

## Supporting Information

Figure S1 Complex epistatic interactions in seeds, flower buds and leaves of *Brassica oleracea*. Epistasis network for all analysed glucosinolates. Red lines indicate epistatic interactions for indolic glucosinolates and black lines for aliphatic glucosinolates (a). Epistasis network for individual glucosinolates. In both panels, dot and solid lines indicate negative and positive epistasis, respectively (b).
(PDF)

Table S1 List of metabolic quantitative trait loci (QTL) for glucosinolates in the three plant organs.
(DOCX)
