# Supplementary figures and images for: Identification of Metabolic QTLs and Candidate Genes for Glucosinolate Synthesis in Brassica oleracea Leaves, Seeds and Flower Buds
Source: PLoS One. 2014 Mar 10;9(3):e91428. doi: 10.1371/journal.pone.0091428 (PMC3948865; doi:10.1371/journal.pone.0091428)

(a)

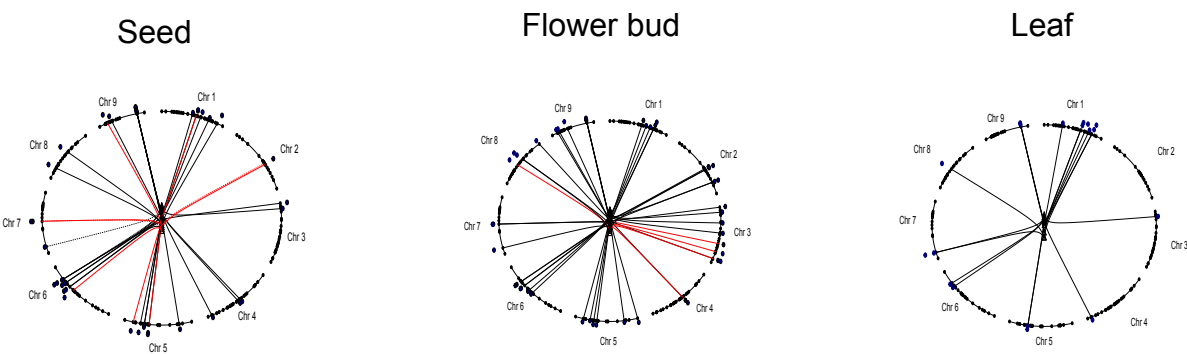

(b)

GBS

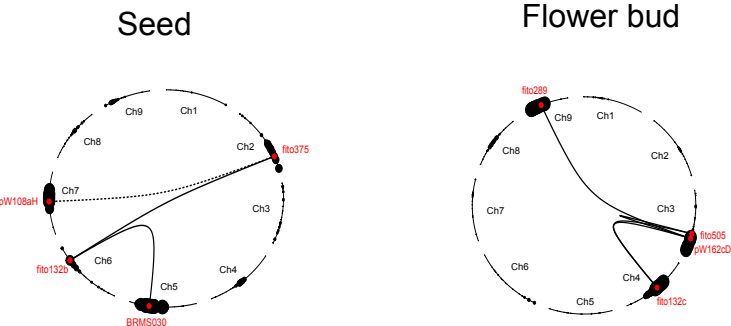

GER

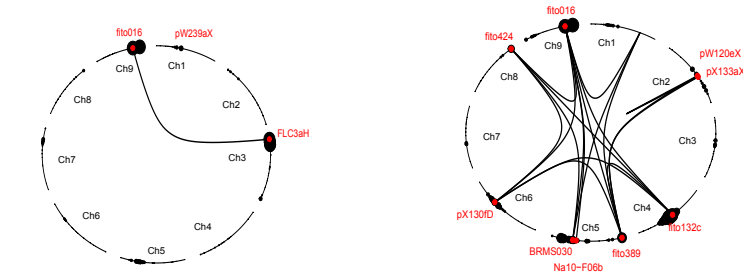

GIB

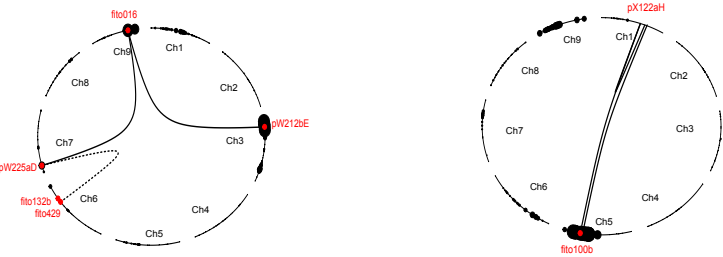

GIV

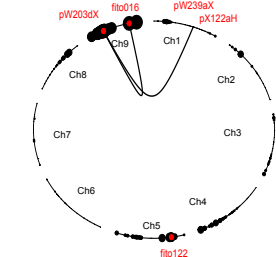

Seed

Flower bud

Leaf

GNA

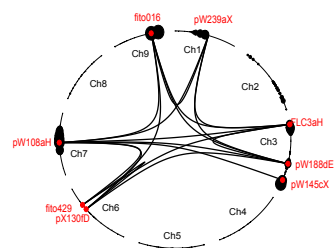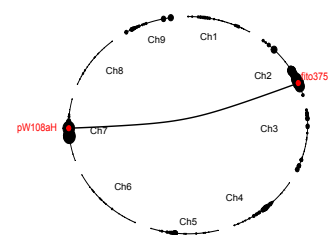

GRA

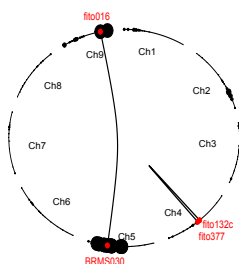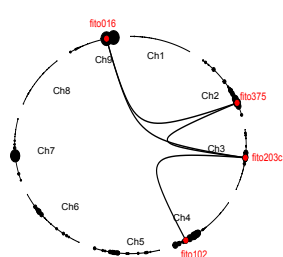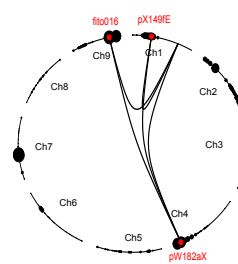

NeoGBS

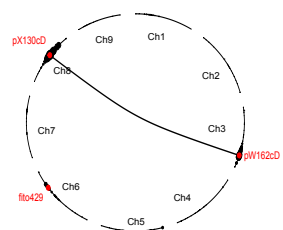

OHGBS

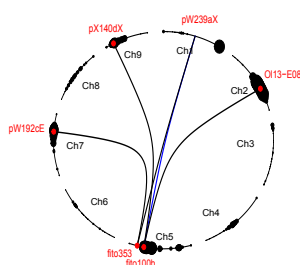

PRO

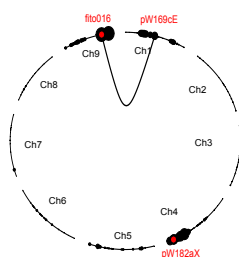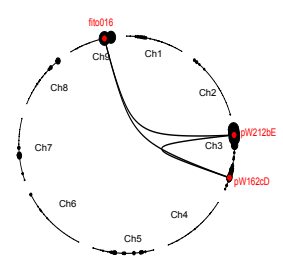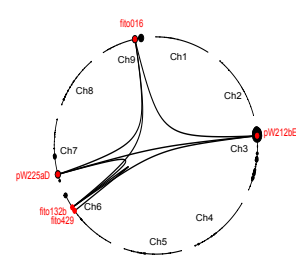

SIN

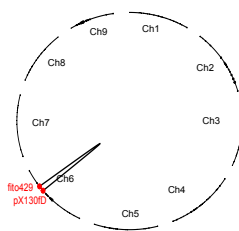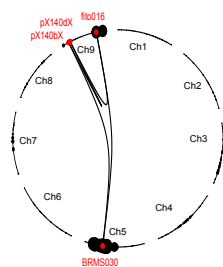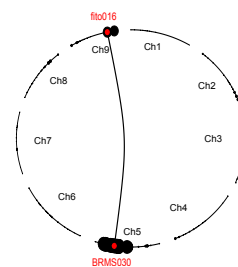

Supplement: Figure S1 — Complex epistatic interactions in seeds, flower buds and leaves of Brassica oleracea. Epistasis network for all analysed glucosinolates. Red lines indicate epistatic interactions for indolic glucosinolates and black lines for aliphatic glucosinolates (a). Epistasis network for individual glucosinolates. In both panels, dot and solid lines indicate negative and positive epistasis, respectively (b). (PDF) [file pone.0091428.s001.pdf]
