# Supplementary material for: Identification of Metabolic QTLs and Candidate Genes for Glucosinolate Synthesis in Brassica oleracea Leaves, Seeds and Flower Buds
Source: PLoS One. 2014 Mar 10;9(3):e91428. doi: 10.1371/journal.pone.0091428 (PMC3948865; doi:10.1371/journal.pone.0091428)
Supplement: Table S1 — List of metabolic quantitative trait loci (QTL) for glucosinolates in the three plant organs. (DOCX) [file pone.0091428.s002.docx]

| **Plant organ** | **Trait** | **Linkage group** | **Peak Position** | **Confidence interval (cM)** | **Left mark** | **Right mark** | **LOD** | **Cross validation frequency (%)** | **Additive effect** | **R^2^%** | **Adj R^2^%** |
| --- | --- | --- | --- | --- | --- | --- | --- | --- | --- | --- | --- |
| Leaves | GIB | 5 | 72 | 70-74 | BRMS030 | BRMS020 | 8.5 | 98.0 | -0.108 | 25.2 | 21 |
|  | SIN | 5 | 57 | 51-63 | pW209aH | fito156a | 6.76 | 99.9 | -0.119 | 20.6 | 20.6 |
|  |  | 9 | 71 | 61-71 | fito017b | - | 3.68 | 39.0 | -0.077 | 11.8 |  |
|  | GRA | 1 | 73 | 72-74 | pW175aX | pW145dX | 4.82 | 63.1 | 0.564 | 15.4 | 24 |
|  |  | 7 | 42 | 40-47 | pW104aE | pW108aH | 5.6 | 30.5 | -0.611 | 17.6 |  |
|  |  | 9 | 65 | 55-71 | fito017b | pW187bH | 5.01 | 99.3 | 0.461 | 15.9 |  |
|  | GNA | 3 | 7 | 5-10 | pW174aX & pW256aH | pW212bE | 3.91 | 45.2 | -0.284 | 11.0 | 25.5 |
|  |  | 7 | 55 | 54-67 | pW108aH | fito088b | 3.64 | 35.5 | -0.268 | 10.3 |  |
|  |  | 9 | 62 | 49-67 | fito016 & fito017b | pW187bH | 9.17 | 100.0 | -0.413 | 23.9 |  |
|  | PRO | 3 | 7 | 5-10 | pW256aH | pW212bE | 5.69 | 97.3 | 0.228 | 17.7 |  |
|  |  | 7 | 42 | 40-48 | pW104aE | pW108aH | 4.18 | 59.9 | -0.166 | 13.3 |  |
|  |  | 9 | 66 | 51-71 | fito017b | pW187bH | 4.59 | 81.4 | -0.178 | 14.5 | 21 |
|  | GNT | 5 | 83 | 79-85 | fito100b | pX119dH | 3.21 | 27.2 | 0.096 | 10.4 | 17.3 |
|  |  | 7 | 2 | 0-12 | fito472 | pW225aD | 3.32 | 19.9 | 0.054 | 10.8 |  |
|  |  | 8 | 44 | 42-51 | fito204a | fito018 | 3.33 | 26.0 | 0.055 | 10.8 |  |
|  | Aliphatic | 2 | 89 | 82-95 | fito375 | fito034 | 4.62 | 75.1 | -1.493 | 14.5 | 35.2 |
|  |  | 3 | 100 | 95-100 | BRMS015a | - | 3.91 | 48.0 | -1.593 | 12.4 |  |
|  |  | 7 | 43 | 41-48 | BRMS042 & BRMS050 | pW108aH | 12.39 | 100.0 | -2.603 | 34.3 |  |
|  | Indolic | 6 | 81 | 80-82 | fito190 | fito429 & pW217cE | 3.61 | 42.1 | -3.693 | 11.5 | 12.4 |
|  |  | 8 | 48 | 45-53 | fito018 | pX130cD | 4.62 | 48.2 | 1.200 | 14.5 |  |
| Flowerbuds | GIB | 5 | 71 | 68-72 | pW164aE | BRMS020 | 12.7 | 95.6 | -0.456 | 34.9 | 33.8 |
|  | SIN | 5 | 69 | 66-71 | pW160dH | pW164aE | 6.3 | 50.3 | -0.333 | 19.2 | 21.2 |
|  |  | 9 | 68 | 60-71 | fito016 & fito017b | pW187bH | 4.1 | 36.2 | -0.266 | 12.9 |  |
|  | GER | 3 | 47 | 45-48 | fito203c | pX111aD & fito040a | 4.2 | 65.3 | 0.048 | 13.3 | 13.3 |
|  |  | 9 | 63 | 50-70 | fito016 & fito017b | pW187bH | 6.0 | 98.6 | 0.040 | 18.4 |  |
|  | GRA | 9 | 61 | 55-65 | fito016 & fito017b | pW187bH | 9.6 | 99.2 | 1.938 | 27.8 | 19.5 |
|  | GNA | 3 | 7 | 5-10 | pW174aX & pW256aH | pW212bE | 3.9 | 60.5 | -1.106 | 12.3 | 41.2 |
|  |  | 3 | 98 | 94-100 | BRMS015a | pX146dH | 5.0 | 47.0 | -1.422 | 15.7 |  |
|  |  | 7 | 58 | 54-66 | pW108aH | fito088b | 5.9 | 68.8 | -1.409 | 18.0 |  |
|  |  | 9 | 60 | 52-65 | pW108gH | pW187bH | 10.3 | 99.2 | -1.717 | 29.5 |  |
|  | PRO | 3 | 7 | 5-9 | pW174aX & pW256aH | pW212bE | 14.5 | 95.8 | 1.664 | 38.8 | 34.2 |
|  |  | 9 | 66 | 52-71 | fito016 & fito017b | pW187bH | 3.6 | 25.4 | -0.622 | 11.5 |  |
| **Plant organ** | **Trait** | **Linkage group** | **Peak Position** | **Confidence interval (cM)** | **Left mark** | **Right mark** | **LOD** | **Cross validation frequency** | **Additive effect** | **R^2^%** | **Adj R^2^%** |
|  | OHGBS | 2 | 66 | 63-69 | pW120eX &fito161 | Ol13-E08 | 4.9 | 73.2 | 0.094 | 15.3 | 12.7 |
|  | GBS | 3 | 72 | 69-74 | fito227 | pW196aH, fito488, pX131bX, pX119bH & pW219aE | 3.4 | 26.9 | -0.668 | 10.8 | 41.1 |
|  |  | 3 | 84 | 83-89 | fito306 | pW145cX | 4.1 | 54.6 | 0.983 | 13.0 |  |
|  |  | 4 | 52 | 50-55 | fito139b | fito132c | 3.4 | 28.6 | 0.449 | 10.8 |  |
|  |  | 9 | 34 | 30-41 | pW108gH | fito016 & fito017b | 6.7 | 99.9 | 0.677 | 20.4 |  |
|  | NeoGBS | 6 | 81 | 80-82 | fito190 | fito429 | 5.5 | 80.1 | -4.073 | 17.1 | 5.9 |
|  |  | 8 | 50 | 45-53 | fito018 | fito146b | 4.0 | 45.4 | 0.915 | 12.7 |  |
|  | GNT | 8 | 47 | 45-53 | fito018 | pX130cD | 7.5 | 100.0 | 0.124 | 22.3 | 25.8 |
|  | Aliphatic | 2 | 90 | 83-95 | fito375 | fito034 | 7.1 | 99.5 | -1.679 | 21.3 | 46.6 |
|  |  | 3 | 46 | 40-47 | pW125dE | pW172aH | 3.3 | 22.1 | 1.167 | 10.4 |  |
|  |  | 3 | 100 | 95-100 | BRMS015a | - | 5.3 | 91.1 | -1.401 | 16.3 |  |
|  |  | 7 | 43 | 41-45 | BRMS042 & BRMS050 | pW108aH | 20.3 | 100.0 | -3.164 | 49.7 |  |
|  | Indolic | 6 | 81 | 80-82 | fito190 | fito429 | 3.8 | 78.0 | -3.995 | 11.9 | 12.2 |
|  |  | 8 | 48 | 45-53 | fito018 | pX130cD | 3.3 | 22.7 | 1.131 | 10.7 |  |
|  |  | 9 | 12 | 10-13 | pW189aX | pX140dX | 3.3 | 37.2 | 2.848 | 10.6 |  |
|  | Total | 2 | 90 | 81-95 | fito375 | fito034 | 3.8 | 43.0 | -1.853 | 12.0 | 14.8 |
|  |  | 7 | 63 | 55-67 | fito088b | pX110aE | 4.4 | 67.0 | -2.312 | 13.9 |  |
| Seeds | GIV | 5 | 66 | 65-67 | fito316, pX147fH, pX126bX, fito259, pW127cE & fito279a | pW160dH | 8.01 | 64.2 | 23.121 | 24.2 | 1.7 |
|  |  | 5 | 84 | 83-85 | fito100b | fito279b & pW213bX | 19.43 | 80.0 | -26.917 | 49.0 |  |
|  | GIB | 5 | 73 | 71-74 | BRMS030 | pW198bH | 15.64 | 99.6 | -6.353 | 41.8 |  |
|  |  | 9 | 66 | 46-71 | fito016 & fito017b | pW187bH | 4.15 | 59.5 | 3.021 | 13.6 |  |
|  | SIN | 5 | 68 | 66-71 | pW160dH | pW164aE | 14.86 | 100.0 | -7.372 | 40.2 | 45.1 |
|  |  | 9 | 21 | 19-22 | pW203dX, pW233aE & fito287a | pX147iH & pX146cH | 4.44 | 67.7 | -3.605 | 14.2 |  |
|  |  | 9 | 70 | 65-71 | fito016 & fito017b | pW187bH | 11.16 | 100.0 | -5.846 | 32.1 |  |
|  | GER | 9 | 67 | 63-71 | fito016 & fito017b | pW187bH | 13.87 | 99.9 | 6.754 | 38.2 | 22.2 |
|  | GRA | 4 | 84 | 78-92 | fito017a | fito102 | 4.04 | 51.6 | -6.794 | 13.1 | 47.5 |
|  |  | 9 | 65 | 61-69 | fito016 & fito017b | pW187bH | 17.99 | 99.8 | 15.862 | 46.4 |  |
|  | GNA | 3 | 6 | 5-9 | pX141bH | pW212bE | 5.33 | 90.5 | -16.807 | 16.9 | 43.9 |
| **Plant organ** | **Trait** | **Linkage group** | **Peak Position** | **Confidence interval**  **(cM)** | **Left mark** | **Right mark** | **LOD** | **Cross validation frequency** | **Additive effect** | **R^2^%** | **Adj R^2^%** |
|  |  | 9 | 64 | 56-68 | fito016 & fito017b | pW187bH | 12.52 | 99.6 | -24.759 | 35.2 |  |
|  | PRO | 3 | 8 | 7-10 | pW174aX & pW256aH | pW212bE | 18.45 | 98.5 | 33.357 | 47.2 | 42.7 |
|  |  | 6 | 90 | 87-98 | fito373b | fito040c | 3.96 | 54.0 | -11.643 | 12.8 |  |
|  |  | 9 | 63 | 46-71 | fito016 &fito017b | pW187bH | 5.32 | 94.0 | -8.228 | 16.2 |  |
|  | ALY | 5 | 84 | 83-85 | fito100b | fito279b & pW213bX | 19.65 | 80.0 | -42.963 | 49.4 | 0.6 |
|  | OHGBS | 5 | 72 | 70-74 | BRMS030 | BRMS020 | 8.02 | 100.0 | -1.899 | 24.3 | 43.1 |
|  |  | 7 | 52 | 45-59 | BRMS042 & BRMS050 | pW108aH | 7.87 | 99.9 | 1.964 | 23.8 |  |
|  |  | 8 | 52 | 45-53 | pX130cD | fito373c | 3.86 | 42.6 | -1.231 | 12.5 |  |
|  | GBS | 1 | 94 | 87-95 | fito426 | pW248aX | 4.40 | 71.5 | 0.563 | 14.1 | 36.7 |
|  |  | 2 | 68 | 64-72 | pW250bH, pWd251hX, pX128dX, pW148aE, fito237 & pW177cH | Ol13-E08 | 9.72 | 100.0 | -0.809 | 28.6 |  |
|  |  | 4 | 44 | 42-48 | pX111eD | pW193bE | 4.00 | 58.9 | 1.569 | 12.9 |  |
|  |  | 5 | 62 | 56-65 | pW209aH | fito156a, pX139dH, pX101aX & pW190cX | 3.58 | 33.9 | -0.765 | 11.7 |  |
|  |  | 5 | 85 | 84-86 | fito294a | fito353 | 10.61 | 78.9 | 1.607 | 30.7 |  |
|  |  | 6 | 94 | 86-105 | fito132b | fito040c | 3.65 | 38.3 | -0.520 | 11.9 |  |
|  |  | 7 | 76 | 74-78 | pW192cE | pX126aX, BRMS040a & B.n.50F | 6.69 | 99.7 | 0.851 | 20.7 |  |
|  | NeoGBS | 5 | 84 | 80-85 | fito100b | fito279b & pW213bX | 4.22 | 68.8 | -0.490 | 13.6 | 10.4 |
|  |  | 8 | 31 | 26-37 | pWd251gBr | pW231aX | 4.66 | 73.3 | 0.261 | 14.9 |  |
|  | Aliphatic | 7 | 43 | 40-48 | BRMS042 & BRMS050 | pW108aH | 4.44 | 66.7 | -2.026 | 14.0 | 28.0 |
|  |  | 7 | 58 | 54-67 | pW108aH | fito088b | 7.93 | 95.8 | -2.544 | 23.6 |  |
|  | Indolic | 8 | 51 | 50-59 | pX130cD | fito146b | 3.21 | 16.1 | 1.133 | 10.3 | 11.8 |
|  | Total | 6 | 92 | 87-99 | fito132b | fito040c | 5.89 | 81.9 | -31.215 | 18.4 | 16.9 |
|  |  | 9 | 66 | 60-71 | fito016 & fito017b | pW187bH | 4.07 | 36.0 | -14.543 | 13.1 |  |

Aliphatic glucosinolates: GIV, Glucoiberverin; GIB, Glucoiberin; Sin, Sinigrin; GER, Glucoerucin; GRA, Glucoraphanin; GNA, Gluconapin; PRO, Progoitrin; ALY, Glucoalyssin; GBN, Glucobrassicanapin; Indolic glucosinolates: OHGBS, 4-hydroxyglucobrassicin; GBS, Glucobrassicin; NeoGBS, Neoglucobrassicin: Aromatic glucosinolate:GNT, Gluconasturtiin. Additive effect was calculated as (P_2_-P_1_) /2; R^2^ %: coefficient of determination of each mQTL. Adj R^2^ %: coefficient of determination of each trait.
